# Essential Oils and Cultural Heritage Conservation: Are They Safe, Environmentally Friendly, Sustainable, and Negligibly Toxic?

**DOI:** 10.3390/gels11120978

**Published:** 2025-12-05

**Authors:** Daniela Pinna

**Affiliations:** Independent Researcher, Bologna, Italy; daniela.pinna@outlook.com

**Keywords:** control of microbial colonization, cultural heritage objects, essential oils, hydrolates, efficiency, toxicity, environmental impact

## Abstract

Microbial colonization of heritage materials is a well-known conservation issue. When necessary, it is removed using mechanical, physical, or chemical methods, with biocide formulations being a common choice. The need to reduce dependence on conventional biocides has led to the exploration of innovative alternative methods and new formulations with biocidal properties for the conservation of heritage objects. Alternative approaches include natural compounds such as plant essential oils. While these natural options show promise, they present challenges—such as inconsistent effectiveness, possible toxicity, and the need for thorough compatibility testing with historic materials. Therefore, although some concerns are legitimate, the “run” to alternative substances is a growing concern as well. A comprehensive selection and examination of international research articles from the past two decades on this subject has been conducted. The detailed and critical analysis of existing data on essential oils, hydrolates, and other plant-derived extracts studied to prevent and/or eradicate the colonization of microbial communities on heritage objects focuses on their effect on microorganisms in controlled environments, in situ applications on microorganisms, encapsulation in hydrogels and emulsions, toxicity and ecological impact, and alterations of heritage materials. The review also discusses the advantages, limitations, and practical implications of these strategies.

## 1. Introduction

The weathering of cultural heritage objects is influenced by a complex combination of physical, chemical, and biological factors. The colonization of heritage materials—such as textiles, paper, wood, and stones—by microorganisms is a well-documented aspect of conservation. When necessary, biological growth is removed using mechanical, physical, or chemical methods, with biocide formulations being a common choice. However, concerns regarding toxicity and environmental impact are significant factors when selecting biocides. Moreover, resistance of biofilms to environmental stressors, including antimicrobials, is emerging as a worldwide concern, affecting various fields.

In recent years, there has been a growing interest in exploring alternatives to conventional biocides used in conserving artworks [1,2]. The term “biocide” has garnered a negative perception among many conservators, primarily due to health and safety concerns [3]. It is worth underlining that biocides are among the most rigorously regulated and controlled chemicals due to their potential effects on human health and the environment. Despite extensive testing and strict regulations before biocides are approved for sale and labeling, concerns about their health effects persist (see review in [4]). However, it is important to note that “biocide” refers to a wide and diverse range of substances, and the term itself does not inherently imply negativity. As the control of biological growth is often necessary, the need to reduce dependence on conventional biocides has led to the exploration of innovative alternative methods and new formulations with biocidal properties for the conservation of heritage objects. Alternative approaches include natural compounds such as plant essential oils. While promising, these approaches face challenges such as variability in efficacy, potential toxicity, and the need for extensive testing to ensure they are compatible with historic materials. Therefore, although some concerns are legitimate, the “run” to alternative substances is a growing concern as well, as it will be further discussed.

Essential oils (hereinafter referred to as EOs) are highly concentrated distillation products of aromatic plants. They are complex mixtures of hydrocarbons, alcohols, aldehydes, esters, ethers, ketones, oxides, phenols, and terpenes produced in distinct parts of plants (flowers, leaves, stems, seeds, fruits, roots, bark) as secondary metabolites [5,6]. More than 90% of global production is utilized by three major industries worldwide: food flavoring, perfumery, and pharmaceutics [2].

EOs are known for their strong antimicrobial and pesticide properties. They are volatile and lipophilic substances, which means they can effectively penetrate cell membranes. Despite a centuries-long history of use, their antimicrobial mechanisms have not been fully elucidated [6,7]. The effectiveness of EOs relies heavily on their quality. As Holmes [7] notes, for an EO to be considered bioactive, it must meet four essential standards: it should have a verified biological identity, be pure, come from intact source plant material, and be obtained through a well-understood extraction process.

Other plant derivatives have been experimented with in the field of cultural heritage conservation. Hydrolate extracts (also known as hydrosol, floral water, aromatic water, or herbal water) are by-products of the steam distillation process of EOs. They are colloidal suspensions composed of a continuous phase, the distilled water, and a dispersed phase, the emulsion of EO droplets and water-soluble components. Their composition can significantly differ from that of the respective EOs [8]. They are much more diluted and have lower antimicrobial activity but they are safer and can be used in situations where EOs are not suitable. Phytocomplexes, which are blends of molecules with a wide range of actions, remain quite unexplored in this field.

Although plant-based products are often viewed as more environmentally friendly and safer than chemical alternatives, there is insufficient scientific evidence to fully support this belief [9]. This highlights the need for broader safety evaluations to better understand their ecological impact. With increasingly strict international regulations concerning the environmental effects of chemicals, chemical mixtures, and new products—such as the REACH regulation in the European Union and the globally adopted GHS by the United Nations—additional research will be hopefully conducted to produce scientific data on the effects of EOs, hydrolates, and other plant-derived extracts as emerging sources of bioactive compounds [9].

**Figure 1:**
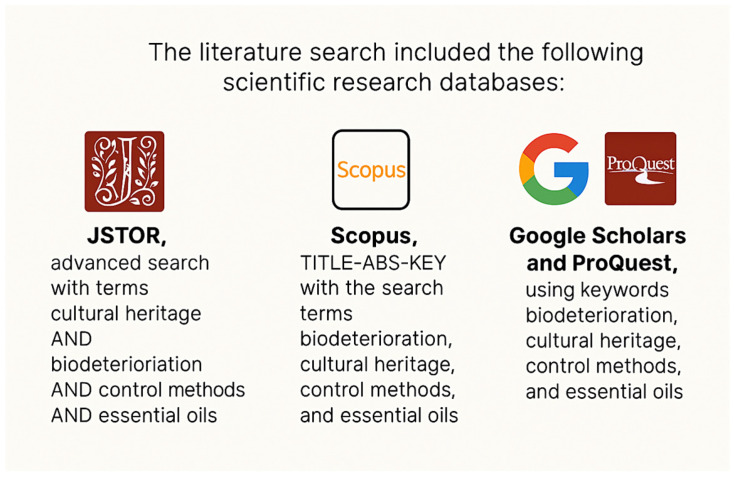
Visual diagram summarizing the literature search strategy across databases.

This review aims to provide a detailed and critical analysis of existing data on plant EOs, hydrolates, and other plant-derived extracts experimented to prevent and/or eradicate the colonization of microbial communities on heritage objects, and it focuses on various related aspects: (i) effect on microorganisms in controlled environments; (ii) in situ applications on biofilms and lichens found on different heritage materials; (iii) encapsulation in hydrogels and emulsions; (iv) toxicity and ecological impact; (v) alteration of heritage materials; (vi) how all this information impacts their use on cultural heritage objects. The review also discusses the advantages, limitations, and practical implications of these strategies, fostering a constructive dialogue between conservation professionals and supporting the development of sustainable approaches to managing biological colonization on stone heritage. To achieve this, a comprehensive selection and examination of international papers from the past two decades on this subject was conducted, as described in Figure 1.

In a later phase, combinations of keywords were used. Additional papers were identified by consulting the reference lists of the selected articles. I would like to highlight the work of Peter Holmes, a medical herbalist and EO therapist. He has authored several renowned textbooks on herbal and EO medicine, including the two-volume series titled ‘Aromatica: A Clinical Guide to Essential Oil Therapeutics’ (2019) [7]. These books have been an invaluable source of information on EOs. Another very important, comprehensive and evidence-based resource on the safety of EOs has been the book ‘Essential Oil Safety: A Guide for Health Care Professionals’ by Robert Tisserand and Rodney Young (2013) [10], which is a landmark reference in the field of aromatherapy and essential oil research.

## 2. Effect of Essential Oils, Hydro-Alcoholic Extracts and Hydrolates on Microorganisms in Controlled Environments

The antimicrobial activity of EOs has primarily been assessed through laboratory experiments using various methods (see Table S1). Common approaches include the disk diffusion test, the tube dilution test, the well plates test and the poisoned food technique.

Regarding the first, Petri dishes containing growth media, microorganisms, and EO-impregnated filter paper disks are incubated under optimal environmental conditions. Regarding the tube dilution test, serial dilutions of the antimicrobial agent are made in a liquid growth medium inoculated with microorganisms and then incubated. The well plates test uses multiple wells as small test tubes. The poisoned food technique is used to evaluate the antifungal activity of EOs by incorporating them into a growth medium for fungi before it solidifies.

A further method adopts the evaporation of EOs instead of direct contact with microorganisms. Filter paper discs soaked with EOs are placed on the lids of Petri dishes where microbial colonies grow on specific media.

The minimum inhibitory concentration (MIC) is determined as the lowest concentration of an EO at which microbial growth is no longer visible compared with the control. Similarly, the Minimum Fungicidal Concentration (MFC) is the lowest concentration of the substance at which no visible fungal growth occurs. 

In certain cases, microorganisms are inoculated onto samples designed to simulate cultural heritage materials (such as wood, paper, stone, wall paintings, and canvas paintings). Treatments are applied not only to bio-colonized surfaces but also to uncolonized surfaces to investigate possible interactions between EOs and the materials themselves.

All these simulated laboratory tests offer significant advantages over in situ studies, as they allow for reproducibility and comparison of results before and after treatment. In other words, they simplify the complexity of nature to make it more understandable.

The target microorganisms of these tests were sampled from artworks made of organic and inorganic materials (paper, wood, canvas, wall paintings, stone, etc.). Most were fungi, a few bacteria (heterotrophic and phototrophic) and algae.

For clarity and ease of reading, I refer to EOs by their common names throughout the text. The taxonomic names of the plants along with the EOs common names are listed in Table 1.

Several EOs are referenced across the reviewed literature, but some—such as EOs of cinnamon, clove, common thyme and oregano—are mentioned more frequently and are recognized for their superior antimicrobial effectiveness also at low concentrations [11,12,13,14,15,16,17,18,19,20,21,22,23,24,25,26,27,28,29]. Also, fennel EO demonstrated good antifungal efficacy [30]. Additionally, some studies have evaluated the individual components of various EOs. Table S1 presents the EOs, the hydrolates, their concentrations, the microbial species they targeted in the in vitro experiments, and the associated references.

It is worth mentioning the contrasting results obtained with Essenzio©, a commercial product composed of oregano EO. Some papers wrongly report that it is a blend of oregano and common thyme [29,31,32,33]. Used to treat phototrophs on wall painting model samples, it showed effective at a dilution of 50% in water [32], while undiluted it was instead ineffective on microalgal biofilms [29]. Quite strange opposite results.

There are EOs with extremely low antifungal activity such as spike lavender, rosemary and basil EOs [24,27,34]. Basil, tea tree, and English lavender EOs resulted almost ineffective against a microalgal biofilm [29], while sea fennel oil showed weak antibacterial activity [18].

It has also been found that the chemical composition of the EO from a plant species can vary. Gas chromatography–mass spectrometry (GC-MS) analysis highlighted compositional differences between basil EO obtained commercially and that produced in the laboratory. The lab-produced oil had higher levels of eucalyptol and α-bergamotene, while the commercial oil contained more linalool [34]. Similarly, in a recent study [35], the chemical composition of oregano EO was found to differ from that reported in previous research. Notably, terpinene, identified by this study as the most abundant compound, was not listed as the dominant component in other studies. According to the authors [35], this variety in chemical composition can be due to the different geographic origins of EOs since the studies have been conducted in more than twenty different countries. Another illustrative example comes from two separate studies: while Arantes and coauthors [36] identified 1,8-cineole as the primary component in calamint EO, Genova and coauthors [37] found this compound present at just 0.5%.

These findings underscore a crucial point: the antimicrobial properties of EOs can vary significantly depending on the production method, which influences their composition, as well as on the geographic origins and age of the plants from which the oils are derived [16]. An additional key point here is related to the chemotype (ct.), that is a group of plants within the same species that differ from others in that species based on the chemical makeup of their secondary metabolites. For instance, *Thymus vulgaris* has many chemotypes, among them ct. thymol, ct. linalool, ct. geraniol, ct. thujanol, ct. paracymene, and ct. cineole. They differ in the dominant constituents and, consequently, in their characteristics. As each EO has a unique chemical profile, the alteration of the proportions of these chemical constituents can result in mixtures with entirely different levels of efficacy. Thus, it is crucial to disclose the full composition of an EO to ensure accurate interpretation of results [38], yet this information is overlooked or insufficiently detailed in some scientific studies.

Studying the effects of English lavender EO on cyanobacteria, a recovery rate of 20% was noticed [39]. According to the authors, this was not due to new growth but rather to the repair of the microorganisms’ photosynthetic structures. This study highlights the initial efficacy of EOs followed by the microorganisms’ ability to restore their structures. Such findings have implications for the required quantities of oils, the frequency of applications, and the resistance of microorganisms to the damaging effects of these compounds—contrary to claims made by many other studies.

Some studies have evaluated the effectiveness of EOs in comparison with conventional biocides, which are listed in Table S2. When compared with the commercial biocide Biotin^®^ T some EOs showed lower antimicrobial effect. It is the case of mastic thyme, pennyroyal, fennel and green lavender EOs that inhibited fungal and bacterial growth 64%, 32%, 30% and 25% less than Biotin^®^ T (1% *v*/*v*) despite their higher concentration (20%) [40]. The comparison with conventional biocides such as benzalkonium chloride and Preventol^®^ RI80, on the other hand, favored basil EO, as larger quantities of the two biocides were required to inhibit the growth of the tested fungal strains [41].

Cinnamon, oregano, and common thyme EOs at a 5% concentration caused a significant reduction in the photosynthetic activity of algal biofilms. This effect lasted for up to two months after treatment and was comparable in effectiveness to biocidal treatments using Bioban^®^ TM 104 Antimicrobial and benzalkonium chloride, both applied at 3% [29].

Calamint EO and its active component pulegone were better than Preventol^®^ RI80 applied on a biofilm composed of green algae and cyanobacteria [37].

A few experiments were conducted on hydro-alcoholic extracts such as that of rosemary [42] or hydrolates such as that of bitter orange [23]. They inhibited the growth of fungi and bacteria, but the quantity needed is far higher than that of EOs—mg/mL vs. µg/mL or quite high concentrations. Some did not even show any antimicrobial activity, as was the case of extracts of mastic thyme, pennyroyal, fennel, and green lavender [40]. Better results were obtained using alcoholic leaf extract of liquorice and a mixture of it with English lavender EO when applied twice on photosynthetic and heterotrophic microorganisms [39]. Stronger antimicrobial activity was performed by garlic extract at different concentrations compared with tea tree EO and calamint extract [43].

The production of hydro-alcoholic and water extracts of basil through three methods resulted in different compositions that had varying levels of antifungal effectiveness [34].

Samples of granite and gneiss, as well as replicas of wall paintings of Etruscan tombs were inoculated with fungi and bacteria, then treated three times with emulsified oregano and clove bud EOs [44]. Stone samples showed a sharp drop in viable cells after the first application, but wall painting samples did not. The differing results could be due to stone’s high porosity, which allowed deeper product penetration, requiring multiple treatments for effectiveness. After two months, wall paintings treated with clove bud oil developed heavy fungal growth, possibly because emulsifiers nourished surviving microbes. Even so, after the first application, the fungi *Cladosporium* sp. and *Penicillium* sp. were detected in all samples, and three treatments were still insufficient to completely eliminate them [44].

### 2.1. Emulsions and Mixtures of Essential Oils

To determine whether combining multiple EOs could enhance their effectiveness, some studies have assessed mixtures of EOs. “Zeylantium green emulsion” (Zege) made of bitter orange hydrolate and cinnamon EO (from bark) was effective on fungi growing on canvas samples [45], while a 1:1:1 mix of oregano, lemongrass, and peppermint EOs in a vapor test outperformed individual oils against fungi on archive papers [35]. However, accurately predicting the antimicrobial effectiveness of EO mixtures is highly challenging due to complex interactions between their chemical components, which may result in synergistic or antagonistic effects [46]. This effect also appears with mixtures of EOs components, as shown by the application of blends of thymol, carvacrol, and pulegone on a biofilm covering a travertine wall [47]. In this case, both positive and negative interactions were observed: while some individual active compounds were more effective than the EOs they naturally occur in, combining these compounds actually reduced their overall effectiveness.

### 2.2. Essential Oils Functionalized on Carrier Materials—Hydrogels

Carrier materials help deliver antimicrobial EOs effectively, as they reduce EOs’ volatility and enhance antimicrobial activity even at low concentrations.

An alginate hydrogel loaded with common thyme EO had the greatest inhibitory effect of photosynthetic efficiency compared with English lavender EO [48]. In contrast, complexation of common thyme EO with β-cyclodextrin did not show important levels of fungal inhibition [49]. Incorporating this EO or thymol into an alginate-calcium chloride hydrogel proved highly effective against cyanobacterial biofilms for up to six months post-treatment [50]. Thymol-loaded in chitosan nanoparticles had a better performance than free thymol against the fungus *Aspergillus niger* [51]. Thymol, carvacrol, and eugenol have been functionalized on β-cyclodextrin and phenazine-based cocrystals, offering a novel approach to managing microorganisms that degrade paper. The phenazine-carvacrol cocrystal was most effective against some fungal species [52].

Among sixteen EO hydrolates added to a gellan hydrogel for paper preservation, only wild bergamot and bitter orange showed fungicidal effects [53].

A biofilm developed on granite blocks and composed of green algae and cyanobacteria, the same species found on a historic granitic building in Santiago de Compostela (Spain), was treated with EOs of oregano, common thyme, calamint, and their respective main active components carvacrol, thymol, and pulegone, with all of them embedded in a hydrogel matrix [38]. Calamint and its active component pulegone proved to be the most effective and yielded similar results, comparable to those of uncolonized granite.

Psyllium and psyllium-alginate beads loaded with cinnamon EO (2%) have been suggested as a means to protect antique books, historical documents, and other cellulosic materials such as paper, wood, and textiles from microbial contamination [54].

A general observation about all these results is that biocides, including EOs, frequently show different behavior in laboratory experiments than they do when applied in in situ conditions when targeting the same microorganisms. One reason for this is that the sensitivity of microorganisms to biocides in laboratory settings is typically much higher than in situ conditions. Another reason is that biocides tend to be less effective against sessile microorganisms, such as those forming biofilms, compared with planktonic ones [26]. Biofilms, with their intricate architecture and dynamic nature, create a physical barrier that shields microorganisms from harmful substances like biocides. Moreover, the response of in situ biofilms is influenced by environmental factors [26].

As a result, the dosage needed to eliminate a biofilm in situ might be significantly higher than the amount required to eradicate isolated organisms in vitro. Therefore, due to the substantial differences between laboratory and field conditions, extrapolating results for practical applications often proves challenging. Nevertheless, such studies are valuable in providing a preliminary assessment of the antimicrobial efficacy of EOs.

## 3. In Situ Studies Using Essential Oils, Hydrolates and Plant-Derived Extracts Applications

### 3.1. Inorganic Materials

#### 3.1.1. Field Trials and Biocide Comparisons

Oregano, common thyme, conehead thyme, and clove essential oils (EOs) have been tested in various in situ experiments targeting biofilms with cyanobacteria, algae, and fungi. While lab studies report positive outcomes, field effectiveness varies depending, among others, on microbial composition, climate, and application method. For example, at Florence Cathedral (Italy), oregano and thyme EOs (2% *v*/*v* in water), alone or combined, showed antimicrobial effects on black biofilms, but Biotin^®^ T (2%) was most effective [26]. Combining EOs did not enhance efficacy.

At Rome’s Non-Catholic Cemetery (Italy), three weekly brush applications of oregano, thyme, conehead thyme, and clove EOs (5% in 30/70 ethanol-water) left 40–70% of cells viable [55]. Preventol^®^ RI80 (3% in water) eliminated a black patina mainly made up of cyanobacteria and some black meristematic fungi, as well as a green patina mostly consisting of green microalgae.

Similarly, on mosaics at Ostia Antica (Italy), eucalyptus, basil, clove, common thyme, pine tree, and tea tree EOs (0.4% *v*/*v*) were applied on cyanobacteria and green algae. Tea tree, pine, and common thyme EOs were most effective among those tested, but all were less potent than chemical biocide Preventol^®^ RI50 (3% *v*/*v*) [31].

The application of EOs on a marble statue colonized by cyanobacteria, fungi, and lichens proved to be very difficult [56]. The treatment with EO blends (one containing oregano, conehead thyme and clove, and the other containing oregano, cinnamon, and clove, all 2.25% *w*/*w*) in agar-agar, Politect^®^, and Carbogel required four applications over two months to remove microorganisms. Mixtures did not outperform single oils, and the restoration process was lengthy and involved applying multiple substances to the stone.

EOs have also been experimented indoors. A recent study [57] evaluated the efficacy of undiluted tea tree and common thyme EOs at disinfecting indoor air. The EOs (10–20 drops ≈ 0.5–1 mL), vaporized in an unventilated area of a church in Spain, reduced airborne fungi and bacteria by 77.3% and 95.0%, respectively, without residue on artworks.

#### 3.1.2. Hydrogels

Embedding EOs in various carrier materials helps improve their efficacy by reducing evaporation, as previously discussed. Hydrogels with oregano, thyme, and calamint EOs, as well as their main phenolic components—thymol, carvacrol, and pulegone (2% *w*/*w*) were applied to granite walls with dark and green biofilms [58]. Combinations and individual ingredients were tested. After one month, hydrogels were easily removed, simplifying cleanup. Oregano-containing treatments worked best and were effective against algae but less so on cyanobacteria, which is likely due, according to the authors, to their higher amount of protective extracellular polymers hindering biocide penetration.

An experiment used similar hydrogels (poly(vinyl)alcohol-borax hydrogels) loaded with thyme EO on slabs of Carrara marble and St. Margarethen stone located outdoors and covered by a dark biofilm [59]. The hydrogel showed broad-spectrum activity against bacteria, fungi and algal microorganisms.

#### 3.1.3. Stabilized Systems and Extracts

In one approach, conehead thyme EO, stabilized with 4% kaolinite by mass [60], was applied to outdoor surfaces of ceramic, marble, and cement grit with green algae and cyanobacteria. The emulsion kept surfaces clean for 4 months. Another approach proposed a release system based on halloysite nanotubes [61]. Areas of outdoor ancient limestone Buddha statues were treated with cinnamaldehyde (5% *w*/*v*)-loaded nanotubes water suspension and after a year microbial growth was greatly reduced.

A 10% liquorice leaf extract provided only short-term reduction in cyanobacterial patina, as the biofilm almost fully regrew within two weeks; however, administering a second treatment led to a 57% decrease in microorganisms [39]. The authors suggest that this lack of sustained effectiveness indicates that a higher concentration of the extract may be necessary for lasting results, although the concentration used was already quite substantial.

#### 3.1.4. Commercial EO Blends

When applied at a concentration of 1.4–1.5% in water, the commercial blend Biotersus^®^ (containing clove 35–37.5%, conehead thyme 27.5–30%, and cinnamon 15–16.5%) effectively removed crustose lichens from stone surfaces, provided that the surfaces were kept well-hydrated [62]. In contrast, using inadequate amounts of the product or failing to fully hydrate the lichens—such as by simply brushing on Biotersus^®^ and only covering with plastic film—resulted in poor removal outcomes.

The same product (1.4% in water) was combined with Psyllium seed shells and applied twice to vertical peperino surfaces colonized by cyanobacteria, algae, fungi, and epilithic crustose lichens [63]. The treated areas were sealed with plastic film for a week, and this process completely removed the biofilms, although some lichen remnants persisted. The authors noted that dehydration was a major concern, as it caused some peeling of the Psyllium support.

When compared to chemical biocides (Biotin^®^ R1 + R2 at 3%, NewDes^®^ 50 and Preventol^®^ RI50 at 5%), the performance of cinnamon EO (0.5%), oregano EO (1%), undiluted Essenzio^©^, and Biotersus^®^ (1.4% in water) was notably less effective [33]. Both Biotersus^®^ and oregano EO failed to reduce microorganism vitality even after multiple applications. In contrast, cinnamon oil and Essenzio^©^ required two brush-on treatments to reach the same biocidal effectiveness that chemical biocides achieved with a single application. All products were applied to marble slabs colonized by cyanobacteria, microalgae, and microcolonial fungi.

#### 3.1.5. Environmental Impact and Recolonization

Recolonization of restored surfaces remains a major challenge in conservation of outdoor artworks.

A study on plastered surfaces of a historic building in Chiavari, Italy, found that although initial biocidal treatment removed a green biopatina, recolonization occurred within months [64]. To assess whether further treatments should be performed, considering their environmental impacts, a Life Cycle Assessment (LCA) compared three biocides: Preventol^®^ RI50 (3 applications by brush), Essenzio^®^ (3 applications by brush; main component: oregano oil) and hydrogen peroxide (1 application). While a detailed explanation of the methodology is beyond the scope here, it is noted as particularly interesting. No biocidal treatments fully removed the patina, which persisted in binder-rich areas. Preventol^©^ RI 50 and hydrogen peroxide reduced biocolonization by over 90%, while Essenzio^©^ achieved about 60% reduction. The LCA results indicated that oregano oil, the main component in Essenzio^©^, had the highest environmental impact across all categories examined, including human health, ecosystem quality, and resource scarcity. The authors emphasize that natural products do not always guarantee a lower environmental impact than synthetic alternatives and suggest that a non-treatment option should be considered, especially when the patina on the recolonized surfaces is primarily epilithic, did not cause cracks or loss of cohesion, and resulted in the surface becoming nearly hydrophobic.

### 3.2. Organic Materials

Common thyme, clove, oregano, and cinnamon are also key essential oils used in the treatment of organic materials such as paper, wood, and canvas.

Common thyme EO (0.75% *v*/*v*) was tested both by spraying and by using impregnated contact sheets on a book cover contaminated with fungi and bacteria [21]. Microbial inhibition proved less effective in the areas treated by spraying than in those covered with contact sheets. This difference is likely due to the paper slowing the evaporation of the oil’s volatile components.

Clove EO (5% and 10% *v*/*v* in ethyl alcohol) was applied to beechwood sawhorses from a museum collection that were contaminated with brown-rot and white-rot fungal growth that was initially reduced, but regrowth occurred after 48 days [65].

Three EOs (cinnamon, wild thyme, and common thyme) were tested as possible alternative biocides for the preservation of waterlogged archaeological wood [14]. A hydroalcoholic solution containing the oils (1%), led to a significant decrease in the vitality of the microorganisms present in the wood and the storage water and demonstrated antimicrobial effects higher than 73%, reaching 99–100% for cinnamon oil.

A wooden sculpture was exposed for 15–20 days to vapors (from 0.5 mL) of oregano and common thyme EOs to eliminate *Aspergillus flavus* colonies present on the surfaces. The treatment was successful, and no recolonization occurred after eight months [66].

A combined approach was chosen to eradicate the bacterial and fungal colonization spread over the surface of an African sculpture made of kapok wood. The object was preliminarily treated with a hydro-alcoholic solution of common thyme EO followed by the exposure to vapor of the same EO in a dedicated chamber [20]. The treatment successfully removed the microbes.

Two historical books exhibiting different levels of microbial contamination were treated with vapors of common thyme EO (10% in dimethyl sulfoxide) that demonstrated stronger bacteriostatic effects (ranging from 12% to 100%) compared to its fungistatic activity (ranging from 0% to 99.3%) and showed biocidal activity against 9 out of 16 microbial strains tested, including 5 fungal strains [67].

An oil painting at the Uffizi Museum in Florence, Italy, was treated on its reverse side by spraying an emulsion consisting of 0.3% (*v*/*v*) cinnamon EO and 99.7% (*v*/*v*) bitter orange hydrolate [23]. A total of 96 sprays were applied to the artwork. To prevent the compounds from dispersing, Melinex^®^ was folded to enclose the painting, forming a sealed container bag using insulating tape. The treatment was allowed to act for 24 h and effectively eradicated the fungal contamination.

## 4. Effects of Essential Oils on Material Characteristics

Assessing color alterations has been the primary analytical approach for evaluating the effects of EOs on both inorganic and organic materials. However, interpreting these analytical findings is challenging, as what constitutes an acceptable change in color is often complex and varies depending on the specific object. Visual assessment, although very effective for qualitative evaluation, lacks precision for quantitative evaluation [68]. It is worth noting that the threshold value considered not perceptible by human eye is ΔE* 3.5 [69]. Therefore, ΔE* values below 4 are generally considered acceptable [70]. The total color difference ΔE* between two measurements is the geometrical distance between their positions in the CIE L*a*b* color space where L* indicates lightness, a* is the red/green coordinate, and b* is the yellow/blue coordinate.

Additional analyses were conducted to evaluate the impact of EO treatments on various material properties. For stone, researchers examined changes in mineralogical composition and water absorption. For cellulose-based materials, such as wood and paper, they assessed oxidation and depolymerization processes. Paper samples were further analyzed for mechanical and structural parameters, including stretch, tensile strength, tensile energy absorption, burst index, fiber integrity, and the morphology of printed areas. Table 2, Table 3 and Table 4 provide detailed findings from studies examining the impact of EOs and other plant-derived substances on material color and other properties. For brevity, only the most pertinent results are discussed in the text.

Some studies have demonstrated that EOs can cause noticeable color changes on various materials, even at low concentrations, while others have found no impact on color at all.

Painted stone samples treated with oregano and clove bud EOs [44] showed significant total color difference (ΔE* > 4) with notable variability depending on the pigment. Charcoal black, hematite, malachite, and cinnabar exhibited pronounced color shifts when exposed to clove oil (Table 2).

**Table 2:** Pigment sensitivity to essential oils.

Pigment	Essential Oil	Concentration (% *v*/*v*)	ΔE* (Color Change)	Effect Description	References
Charcoal black, malachite, cinnabar	Clove	7.5	>4	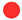 Pronounced color shift	[44]
Cinnabar	Oregano, clove, common thyme	1–10	<2	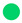	[24]
Hematite	Clove	1–10	2.99	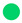	[24]
Hematite	Clove	7.5	>4	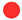 Pronounced color shift	[44]
Hematite	Oregano	1–10	2.89	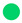	[24]
Hematite	Cassia	1–10	4.06	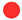	[24]
Hematite	Common thyme	1–10	3.37	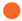	[24]
Oyster shell white	Common thyme, oregano	1–10	<1	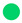	[24]
Oyster shell white	Cassia	3–10	5.89	Yellowing effect	[24]
Vinyl blue	Zeylantium green emulsion	Undiluted	10.3	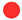 Significant color variation	[45]
Alkyd blue	Zeylantium green emulsion	Undiluted	3–4	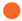 Moderate shifts in unaged samples only	[45]
Acrylic blue	Zeylantium green emulsion	Undiluted	<3	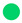	[45]
Alkyd yellow	Zeylantium green emulsion	Undiluted	12.5	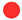 Noticeable shifts in unaged samples only	[45]
Acrylic and vinyl yellow	Zeylantium green emulsion	Undiluted	<2	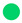	[45]
Acrylic, vinyl, alkyd red, and green	Zeylantium green emulsion	Undiluted	<3	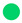	[45]

Color legend: 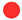 ΔE* > 4 = significant color change, 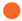 ΔE*~3–4 = moderate change, 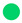 ΔE* 1–3 = minimal change.

In another study, cinnabar, hematite, and oyster shell white pigments mixed with 3% Hide Glue^©^ were sprayed with oregano, clove, common thyme, and cassia EOs [24]. Cinnabar showed slight changes with oregano, clove, and common thyme; oyster shell white remained stable with thyme and oregano but yellowed with clove and cassia (the latter caused strong alteration). Hematite was most affected by cassia EO, followed by thyme, clove, and oregano (Table 2) [24]. Notably, there are conflicting outcomes in these two studies regarding clove EO on cinnabar pigment: while Isola and coauthors [38] reported significant color changes, Lee and Chung [24] found only minor alterations. This discrepancy likely stems from differences in the chemical composition of the clove EOs used in each study.

The application of Zeylantium green emulsion (Zege) (see Section 2.1) to painted canvas samples [45] induced significant color variation in vinyl blue across both unaged and artificially aged specimens. In contrast, alkyd blue, ocher, and red pigments exhibited noticeable shifts exclusively in unaged samples, suggesting a differential sensitivity to Zege based on pigment composition and aging state. Acrylic, vinyl, and alkyd red and green pigments, and acrylic and vinyl yellow pigments did not show significant color changes (Table 2).

Fennel, pennyroyal, and green lavender EOs as well as the biocide Biotin^©^ T (1% *v*/*v*) were applied four times to limestone and calcareous tuff samples [40]. Notably, green lavender caused the most visible changes on limestone—blurring, staining, and reduced lightness—while fennel and pennyroyal had milder effects (Table 3). The commercial biocide Biotin^©^ T did not cause any noticeable changes in color or tone. However, blurring or staining was observed only on samples with very low porosity, likely due to nonvolatile EO residues remaining on the surface; in porous rocks like tuff, these components penetrated deeper, making changes less detectable [40].

**Table 3:** Effects of essential oils and biocides on stone materials.

Stone	Essential Oils and Biocides	Concentration (% *v*/*v*)	ΔE*/ΔL*/Δb* (Color Change)	Effect Description	References
Limestone	Green lavender	20	ΔE* 9.4 ΔL* −9.3	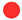 Blurring, staining, marked lightness reduction	[40]
Limestone	Fennel	20	ΔE* 2.3 ΔL* −2.2	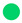 Staining, lightness reduction	[40]
Limestone	Pennyroyal	20	ΔE* 3.4 ΔL* −3.3	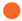 Staining, lightness reduction	[40]
Limestone	Common thyme	0.075	ΔE* 2.5	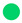 Cumulative risk with repeated treatment	[15]
Limestone	Clove	0.1	ΔE* 0.8	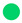	[15]
Limestone	Geranium	0.1	ΔE* 4.1	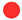	[15]
Limestone, tuff	Biotin^©^ T	1		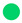 No noticeable color change; staining only on low-porosity rock	[40]
Granite	White thyme	2	ΔE*~6	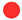	[71]
Schist, mortar, granite	Oregano, common thyme	2		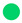 No mineralogical or color changes	[71]
Granite, gneiss	Oregano, clove, common thyme, cassia	1–10		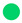 No color or chemical changes	[24]
Granite, gneiss	Oregano, clove	7.5		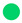 Negligible color change; no change in water absorption	[24,44]
Carrara marble	Oregano, common thyme, Biotin^©^ T	2	ΔE* < 3	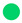 Combination of the two EOs (ΔE*~3); stone yellowing observed	[26]
Brick, peperino, mortar	Nanocomposite with oregano EO or eugenol	—	ΔE* < 3	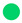 Surfaces hydrophobic, vapor-permeable; no aesthetic alteration	[72]
Peperino	BioTersus^©^	—	—	No color interference	[64]
White sedimentary rock	Cinnamon, oregano	0.5–1	ΔE* < 2 Δb* < 2	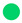 Possible yellowing from Δb*	[33]
White sedimentary rock	Biotersus^©^, Essenzio^©^	1.4 and undiluted	ΔE* < 2 Δb* < 2	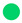 Possible yellowing from Δb*	[33]
White sedimentary rock	Biotin^©^ R1 + R2, NewDes^©^ 50, Preventol^©^ RI50	3/5	ΔE* < 2 Δb* < 2	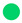 Possible yellowing from Δb*	[33]

Color legend: 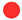 ΔE* > 4 = significant color change, 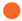 ΔE*~3–4 = moderate change, 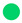 ΔE* 1–3 = minimal change.

EOs of oregano, common thyme, and white thyme were tested on schist, mortar, and granite from Lugo’s Roman wall, Spain. Only white thyme altered granite color; the others caused no mineralogical or color changes [71]. Similarly, oregano, common thyme, clove bud, and cassia EOs had no visible or chemical effects on granite and gneiss. Water absorption remained unchanged after treatment with oregano and clove bud [24,44] (Table 3).

Specimens of white Carrara marble treated with oregano and common thyme EOs, their combination, and Biotin^®^ T showed minimal porosity changes, but common thyme and the blend caused noticeable color shifts (ΔE ≈ 3) after two years of outdoor exposure (Table 3) [26]. Yellowing was observed on Florence Cathedral marble four months after treatments, which is an undesirable side-effect [26].

Silica nanocapsules with oregano EO or eugenol were incorporated into a multifunctional, hybrid nanocomposite coating and applied to samples of brick, peperino, and mortar [72]. The treatment enhanced hydrophobicity while preserving vapor permeability, confirming the effectiveness of the nanocomposite coatings for protecting stone materials from biodeterioration. Additionally, for all treated stones, the measured ΔE values were below 3, proving that the coating maintained the stones’ original appearance and was aesthetically suitable.

Tests on white sedimentary rock with cinnamon bark and oregano EOs, EO-based products (Biotersus^©^, Essenzio^©^), and chemical biocides (Biotin^©^ R1 + R2, NewDes^©^ 50, Preventol^©^ RI50) showed ΔE* < 2 [33]. However, Δb* < 2 for some products (Preventol^©^, Biotersus^©^, Essenzio^©^, and cinnamon oil) suggests potential yellowing with repeated use [33] (Table 3).

Limestone immersed for 8 h in thyme, clove, and geranium EO solutions showed ΔE* of 2.5, 0.8, and 4.1, respectively—geranium caused the most discoloration [15] (Table 3). However, even moderate ΔE* values may pose risks, as repeated applications may cause significant discoloration.

Linalool is a naturally occurring terpene alcohol commonly extracted from lavender, rose, basil, thyme and neroli EOs. In an in vitro experiment, exposure to its vapor caused damage to both silver–gelatin photographic samples and bookbinding leathers [73]. When exposed to air, it becomes unstable and may oxidize into linalool hydroperoxide, a compound known to degrade silver-based photographic images and alter collagen, making it unsuitable for use in storage areas housing these materials [73].

Clove and lavender EO vapors caused significant color changes in gelatin prints and induced oxidation and depolymerization of cellulose in wooden substrates, altering crystallinity [74] (Table 4). A hydro-alcoholic pretreatment with common thyme EO followed by vapor exposure led to moderate color changes (ΔE* 3.82 in light and 2.75 in dark areas) on kapok wood sculptures [20].

**Table 4:** Effects of essential oils and hydrolates on organic materials.

Essential Oils	Material	Treatment Method	Concentration	ΔE* (Color Change)	Observed Effect	Reference
Clove, lavender	Gelatin prints	Vapor exposure	Undiluted	~5	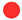 Oxidation and depolymerization of cellulose	[74]
Clove, lavender	Wood	Vapor exposure	Undiluted	<4	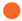 Oxidation and depolymerization of cellulose	[74]
Common thyme	Pine wood	Immersion in solution	0.75 µL/mL	2.7	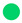	[15]
Clove	Pine wood	Immersion in solution	1 µL/mL	4	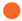	[15]
Geranium	Pine wood	Immersion in solution	1 µL/mL	9	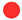	[15]
Common thyme	Historical book	Vapor exposure	10% in dimethyl sulfoxide		Improved mechanical properties; increased bulk	[67]
Wild bergamot, bitter orange hydrolates	Paper from 18th-century books	Hydrogel treatment		1.27 (bergamot), 0.46 (orange)	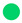 No fiber damage	[53]
Rosemary, lavender	Historical archive paper	Fumigation	1% *v*/*v* (rosemary), 0.4% *v*/*v* (lavender)	0–2.5	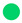	[75]
Common thyme, sage	Cotton and hemp fabrics	Vapor exposure with	Undiluted EOs encapsulated in ethyl cellulose		Thyme decreased strength; sage increased cotton strength but decreased hemp strength	[76]
Cinnamon	Cotton, linen, silk fabrics	Vapor exposure	Undiluted	0.84 (cotton), 2.42 (linen), 2.67 (silk)	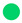 No change in optical, mechanical, or structural properties	[12]

Color legend: 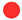 ΔE* > 4 = significant color change, 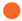 ΔE*~3–4 = moderate change, 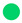 ΔE* 1–3 = minimal change.

Artificially aged pine wood samples immersed in solutions of common thyme, clove and geranium EOs showed the highest ΔE* with geranium oil, indicating strong interaction with the substrate [15] (Table 4).

In paper conservation, thyme EO vapor improved mechanical properties (e.g., tensile strength, burst index) and increased bulk in historical book paper, without altering pH or intrinsic viscosity [67]. Samples from eighteenth-century printed paper treated with gellan hydrogel loaded with wild bergamot and bitter orange hydrolates exhibited minimal color change and preserved fiber morphology [53] (Table 4).

Fumigation with rosemary and lavender EOs reduced fungal colonization on archival paper; lavender EO successfully eliminated the fungus *Trichoderma longibrachiatum* after rosemary failed [75].

Exposure of cotton and hemp fabrics to saturated vapors of common thyme and sage EOs encapsulated in ethyl cellulose altered mechanical behavior: sage EO increased tensile strength in cotton (up to +39%), while thyme EO reduced it in both cotton (−29.9%) and hemp (−40%) due to restricted fiber mobility from microcapsules [76]. In contrast, vapor-phase treatment with pure cinnamon EO did not affect the optical, mechanical, or structural properties of artificially aged cotton, linen, and silk textiles (sericin removed) [12] (Table 4). However, cinnamon EO’s solvent properties caused damage to plastic components (e.g., pumps, accessories) during trials on waterlogged archaeological wood, highlighting potential material incompatibility in conservation setups [14].

## 5. Toxicity, Ecotoxicity and Sustainability of Essential Oils

Many authors in the field of heritage conservation emphasize the low toxicity and minimal environmental impact of essential oils (EOs), often portraying them as sustainable and safe alternatives to conventional chemical biocides. However, it is noteworthy that these claims are frequently presented without direct reference to supporting studies.

The toxicity of EOs is desirable when the goal is to eliminate bacteria, algae, or fungi, and these microorganisms and human cells share some characteristics. So, it should not be totally surprising that some of the most useful antimicrobial EOs possess a degree of human toxicity [10]. It is unrealistic to consider EOs safe and harmless merely because they are naturally derived from plants. There is actually scientific evidence supporting their toxicity and ecotoxicity [77].

### 5.1. Human Toxicity

Some papers [58,78] claim that EOs are classified as “Generally Recognized As Safe” (GRAS) by the US Food and Drug Administration (FDA https://www.fda.gov “URL accessed on 5 November 2025”). This is not correct. Firstly, just some EOs are designated as GRAS—Table 1 lists EOs derived from asterisk-marked plants that are designated as GRAS. Secondly, the GRAS designation indicates that a chemical or substance added to food is considered safe by experts under the conditions of its intended use. In addition, another US regulation, California Proposition 65 (https://oehha.ca.gov/proposition-65/proposition-65-list “URL accessed on 5 November 2025”) restricts the use of certain substances in consumer and lists them on the Proposition 65 List. EOs may contain some of those restricted substances. For instance, beta-myrcene and pulegone, found in EOs, are both listed because they might cause cancer [79,80] (Table 5). Pulegone was used in reviewed papers that claim it is harmless for the environment and human health [37,47,58].

In an effort to demonstrate the safety of hydrolates derived from *Citrus aurantium* and *Monarda fistulosa*, Di Vito and coauthors [53] report that these plants are included in sources permitting their use in human formulations. Regarding *M. fistulosa* they cite the Plant Guide Database of the U.S. Department of Agriculture that provides standardized information about the vascular plants, mosses, liverworts, hornworts, and lichens of the United States and its territories, but does not list plants permitted for use in human formulations, as the authors claim. It is important to note that *Monarda fistulosa* is not listed as “Generally Recognized As Safe” (GRAS) by the U.S. FDA. In contrast, *Citrus aurantium* is currently classified as GRAS (Table 1).

As noted earlier, Peter Holmes’ books [7] provide extensive and reliable information regarding EOs safety in relation to pharmacology and toxicity. He affirms that “their clinical uses have too short a history, their applications are too varied, and the current dichotomy between clinical experience and scientific research is too great.” This indicates that it is incorrect to claim that EOs are safe for humans, as many studies on cultural heritage applications assert. Some EOs pose acute toxicity risks to humans and animals in small doses due to the presence of certain highly toxic components [81]. For example, EOs from *Artemisia absinthium* and *Thuja occidentalis*, both of which contain the neurotoxin thujone, and *Mentha pulegium*, which contains pulegone, another neurotoxin [82] (Table 5).

EOs of clove bud, *Pimenta* berry and leaf, cinnamon leaf, oregano, conehead thyme, chemotypes of *Thymus vulgaris* and winter and summer savory belong to the category of strong skin irritants because of their high content in various phenols, including eugenol, thymol and carvacrol [7] (Table 5). Cinnamon bark and cassia bark oils are strongly irritating to the skin because of their high levels of cinnamic aldehyde. While most EOs do not cause sensitization, the ones mentioned above are known to pose a potential risk for it [7].

Studies investigating acute, developmental, and reproductive toxicity, as well as mucous membrane irritation, of rosemary, citrus, and eucalyptus essential oils have employed both in vitro and in vivo models [81]. Findings showed that all three essential oils had comparable effects on the endpoints examined, with rosemary oil exhibiting slightly greater toxicity. Importantly, significant toxic effects were observed for all oils, even at low doses.

Important studies have highlighted results obtained by experimenting with single components of EOs. Alkenyl phenols exhibit genotoxic and carcinogenic properties [83]. Thymol, 8-cineole (also known as eucalyptol), (αβ)-pinene and limonene induced cytotoxicity and genotoxicity through oxidative damage in diverse types of target cells (see review in [79,84]) (Table 5).

Non-carcinogenic components may also pose concerns such as potential liver, nerve, reproductive, kidney toxicity, and endocrine disruption [83].

Terpenes are a large group of volatile unsaturated hydrocarbons found, among others, in the EOs of plants. They are widely used as food additives and in the medical field. Most terpenes, particularly monoterpenes, have high cytotoxic potential shown in various model organisms, while some of them, such as β-caryophyllene, show anti-inflammatory, antioxidant, and cytoprotective effects [85]. Terpinolene, α-terpineol, humulene and linalool are considered among the most toxic terpenes [85,86] (Table 5). The cytotoxic effect is primarily caused by plasma membrane disruption, lipid peroxidation, reactive oxygen species (ROS) production, mitochondrial transmembrane potential loss, and mitochondrial impairment [85].

Phenolic compounds like carvacrol, thymol, eugenol, and vanillin are widely used in food and other products and are generally considered safe. Significant biological properties, including antimicrobial, antioxidant, analgesic, anti-inflammatory, anti-mutagenic, or anti-carcinogenic activity, have been described for these substances. However, research has found that carvacrol, thymol, and eugenol can have toxic effects, including oxidative stress, mutagenicity, genotoxicity [87,88] and hepatic toxicity [79] (Table 5). In vivo studies show adverse effects after acute and prolonged carvacrol and thymol exposure to mice, rats, and rabbits. Eugenol has caused pulmonary and renal damage to exposed frogs. In humans, these three compounds may cause skin reactions, inflammation, ulcer formation, and slow healing. Vanillin may reduce cell viability at high concentrations [89,90] (Table 5). According to the authors, increased exposure to these substances warrants further safety review for both human and environmental health.

Terpenoids and ketones are associated with neurotoxicity and abortive properties; monoterpenoids are associated with renal toxicity; pinene derivatives, trans-anethol, sclareol or alpha-humulene having hormone-like structures raise the risk of endocrine disruption; and furocoumarins have phototoxic properties [91].

It is worth examining some characteristics of individual EOs, focusing on those that have been most frequently evaluated in the preservation of cultural heritage. The primary references are the books by Holmes [7] and Tisserand & Young [10], with various other sources cited throughout the text.

- Basil is a mild skin irritant.
- Cinnamon bark oil is known for being highly irritating and sensitizing to the skin and mucous membranes due to its components cinnamaldehyde and eugenol.
- Clove bud is a mild skin irritant. It contains 60–96% of phenols including methyl eugenol that despite being moderately toxic, has been demonstrated to be genotoxic and carcinogenic according to The National Toxicology Program of the US Department of Health and Human Services [92] (Table 5). Since 2021, under the Regulation EC No 1272/2008, any product sold in the European Union that contains more than 0.01% methyl eugenol is required to carry a label indicating its presence.
- Oregano is highly dermocaustic (skin irritant). Much commercial oregano oil is extracted from *Thymus capitatus*.
- Common thyme (*Thymus vulgaris* L.). Its key constituents include the phenols thymol and carvacrol, glycosides, flavonoids, p-cymene, borneol, linalool, alcohols, rosmarinic acid, saponins, tannins, and terpenoids [7]. Thyme ct. linalool, thyme ct. geraniol and thyme ct. thujanol are terpenol-dominant. They do not irritate the skin, making them more broadly applicable and versatile in practical use compared to other chemotypes. Terpenols, such as geraniol, linalool, and citronellol, are acyclic compounds, which contain an alcohol functional group (-OH) attached to a monoterpene structure. Geraniol, one of the most important molecules used in cosmetic industries for its antimicrobial activities, can induce allergic reactions such as irritant contact dermatitis (see review in [93]). Thyme ct. thymol is phenol-dominant and is highly irritant to the skin and mucosa. Thymol, a terpenoid phenol, is naturally found in the EO of thyme and various species of the genera *Origanum*, *Satureja*, and many others. It induced cytotoxicity and genotoxicity through oxidative damage in several types of target cells (see review in [84]) (Table 5). *Thymus capitatus* EO is particularly rich in carvacrol-typically ranging from 40–50% and occasionally reaching concentrations as high as 74%. It presents a significant risk of cumulative toxicity and is associated with moderate to severe skin irritation and sensitization [7] (Table 5). Thyme EO has an LD_50_ of 980 mg/kg body weight (oral, rat) [90] (Table 5). Under the European Union Classification Criteria for Acute Toxicity, it corresponds to substances categorized as “harmful if swallowed.”
- Sage contains the toxic substance thujone see review in [79,94] (Table 5).

The study by Nematollahi and coauthors [95] emphasized the need to consider the potential impact on people of volatile organic compounds (VOCs) from EOs. It found that a variety of commercially available EOs with therapeutic claims—including tea tree, lavender, eucalyptus, geranium, peppermint, bergamot, orange, and blended oils—emit VOCs such as acetaldehyde, alpha-phellandrene, alpha-pinene, camphene, limonene, methanol, terpinolene, 3-carene, acetone, β-phellandrene, ethanol, and γ-terpinene, with many present in over 90% of the oils. The most common potentially hazardous VOCs included acetaldehyde, limonene, methanol, acetone, ethanol, 3-carene, and toluene. Each tested EO released at least nine potentially hazardous VOCs, yet less than 1% of all identified VOCs and hazardous VOCs were disclosed on product labels, safety data sheets, or websites.

**Table 5:** The table lists several main components of EOs reported in the reviewed papers and provides various toxicity-related details, including results from both in vitro studies (using cell lines) and in vivo studies (using living organisms). Acronym legend: LD_50_, lethal dose needed to cause death in 50% of a test population over a set time; IC_50_, concentration at which a substance inhibits a chemical or biological process by 50%; NOAEL, no-observed-adverse-effect level; TC_50_, concentration of a substance at which 50% of cell viability is compromised; ECHA, European Chemicals Agency; EMA, European Medicines Agency; IARC, International Agency for Research on Cancer; FDA, Food and Drug Administration; GRAS, Generally Recognized as Safe.

Substance	LD_50_ (mg/kg bw)	NOAEL (mg/kg bw/day)	IC_50_/Cytotoxicity Thresholds	Genotoxicity/Cytotoxicity Observations	Regulatory Classification	Reference
α-terpinene	~1650 (oral, rat)	-	0.5–1.0 mL/kg (daily oral administration rat for 10 days)	Neurotoxicity, oxidative stress, genotoxicity in liver	ECHA: acute Tox. 4; H302—harmful if swallowed	See review in [79]
Carvacrol	810 (oral, rat)	-	≥500 µM L^−1^ (cytotoxicity); ≥115 µM L^−1^ (mutagenic)	Genotoxic at 460 µM L^−1^; apoptosis at 50 mg/L; mutagenic at 115–230 µM L^−1^; marked decreases in cell viability at 500 μM L^−1^	ECHA: Skin corrosive Cat. 1B/C	See review in [88,89,90]
Citral	~5000 (oral, rat)	-	>60 mg/kg	Maternal and embryo toxicity (human body)	EMA: reproductive toxicity concern	See review in [79]
Estragole	~1000–2000 (oral, rat)	-	≥1 mM (estragole); ≥25 µM (1′OH-estragole)	DNA adducts, genotoxicity, cytotoxicity in human liver cells	EMA: genotoxic concern; not formally classified by IARC	[96]
Eugenol	>2000 (oral, rat)	300	~0.75 mM L^−1^ (IC_50_); 750 µM L^−1^; 2500 µM L^−1^; 0.06%	DNA strand breaks, chromosomal aberrations, apoptosis	FDA: GRAS; not acutely toxic	See review in [89,90]
Limonene	4400–5600 (dermal, rat)	-	≥100 µM	Highly toxic to human lung cells	FDA: GRAS; ECHA: sensitizer	See review in [79]
Linalool	~2790 (oral, rat); >2000 (dermal, rabbit)	-	≥0.3% (patch test)	Skin sensitization	ECHA: sensitizer; FDA: GRAS	[86]
Methyl eugenol	~850 (oral, rat)	None (carcinogenic)	10–100 mg/kg/day by gavage (14-week study), rats/mice	DNA adducts, liver tumors; genotoxic and carcinogenic	IARC: Group 2B; FDA: not authorized; EMA: restricted	[92]
Pulegone and menthofuran (product of metabolic pathway of pulegone)	470 (oral, rat); 3090 (dermal, rabbit)		25 ng/mL (pulegone); 41 ng/mL (menthofuran)	Hepatotoxicity, reproductive toxicity, oxidative stress	IARC: possibly carcinogenic to humans, Group 2B; FDA: not authorized as flavoring; EMA: limit 0.1 mg/kg/day	See review in [79,80]
Safrole	~1950 (oral, rat)	-	≥125 µM; TC_50_: 361.9 µM (24 h), 193.2 µM (48 h)	DNA damage, micronuclei formation; genotoxic in vitro and in vivo	IARC: possibly carcinogenic to humans, Group 2B; FDA/EMA: banned in food	[97]
Thujone	~192 (oral, rat)	~5 (rats)	≥25 mg/kg (intraperitoneally for 14 days, mice)	Organ damage, mortality, hepatic and renal dysfunction	EMA: limit 0.1 mg/kg/day; FDA: not authorized as flavoring	See review in [79,82]
Thymol	980 (oral, rat)	667 (subchronic exposure, rat)	~400–700 µM (IC_50_); ≥75–600 µM (apoptosis)	Apoptosis in HL-60 and glioblastoma cells; no cytotoxicity in neurons; no genotoxicity ≤ 250 µM	FDA: GRAS	See review in [88,89,90]
Vanillin	3978 (oral, rat)	650	mM range	Low cytotoxicity; viability reduction at high concentrations	FDA: GRAS; low toxicity profile	See review in [89,90]

### 5.2. Environmental Toxicity and Regulatory Considerations

The potential toxic effects of EOs and plant extracts on living organisms and ecosystems have been poorly investigated [98]. Recently, some studies have started to focus on non-target organisms, which include all living organisms other than those at which the treatment is directed. The active substances in plant EOs can be harmful to phytoplankton, zooplankton, algae, aquatic vertebrates, soil microorganisms, terrestrial vertebrates, and plants [9]. While EOs and plant extracts are generally considered eco-friendly and safer than synthetic alternatives, some of them have been found to be toxic to non-target organisms [9,82]. Currently, data on the effects of plant-based products are still limited, with most studies concentrating on aquatic systems and, in particular, on *Daphnia magna*. The potential impact on other aquatic organisms, as well as marine and terrestrial systems, remains largely unknown.

Another environmental concern related to EOs is their typically low yield from plants, which usually range from 0.1% to 6% by weight depending on the plant species and the part utilized [99]. Although extraction techniques are crucial for obtaining these oils, they often demand large quantities of plant material and considerable energy. This underscores the resource-intensive nature of EO production, which, if not properly managed, can result in overharvesting and the depletion of plant resources [99].

Ferraz and coauthors [9] anticipate that with the tightening of international regulations, such as REACH (Registration, Evaluation, Authorization and Restriction of Chemicals) in the European Union and the globally implemented GHS (Globally Harmonized System of Classification and Labelling of Chemicals) by the United Nations, there will be an increase in studies producing scientific data on the effects of EOs, hydrolates, and other plant extracts as new sources of bioactive compounds.

## 6. Current Limitations and Future Prospects of Essential Oil Applications in Cultural Heritage Preservation

The use of EOs, plant extracts, and hydrolates in cultural heritage conservation is a fascinating yet highly debated area. The literature review highlights the enthusiasm with which many studies report the use of these substances. It is as if a long-sought panacea has finally been found for the biodeterioration of cultural heritage objects. Summarizing some of the most cited claims, these natural substances are portrayed as harmless to artifacts, environmentally sustainable, and safe for restorers. Such affirmations appear somewhat misplaced, given the highly contentious nature of the field. The complexity and diversity of factors involved make it exceedingly difficult to draw consistent or generalizable conclusions. Nonetheless, some critical concerns have emerged—including safety, environmental toxicity, and their potential to foster bio-resistance [38,100,101].

EOs are chemically complex mixtures, often containing dozens of constituents, and it remains challenging to determine which specific components exhibit antimicrobial activity, or whether such effects arise from synergistic interactions between them. The antimicrobial activity of EOs can differ significantly. The same EO has contrasting effects on microorganisms, as oregano EO on algal and cyanobacterial biofilms. This variability can be attributed to differences in chemical composition resulting from distinct extraction techniques, geographical locations, or the specific plant parts from which they originate. Many active components are volatile organic compounds with limited duration of action, complicating both their management and overall efficacy. This inconsistency raises questions about their reliability. Understanding both the production methods and chemical composition of EOs is an important aspect that is frequently overlooked or insufficiently detailed in some scientific studies. Additionally, research highlights that the antimicrobial activity of EOs is influenced by the particular microbial species being targeted. One example is the commercial product Biotersus^®^, which demonstrated fluctuating levels of effectiveness. Another example is the failure of oregano and clove bud EOs at eliminating fungi of the genera *Cladosporium* and *Penicillium* [44].

A relevant point highlighted in the literature is the ability of microorganisms to recover. Three studies [35,39,44] reported an initial reduction in viable cells followed by the ability to repair and restore their structures. Such findings have implications for the required quantities of EOs, the frequency of applications, and the resistance of microorganisms to the damaging effects of these oils.

Some publications also suggest that these substances are environmentally friendly because they are biodegradable. While plant-derived compounds are generally considered readily biodegradable due to their volatility, there is still a notable lack of research investigating their persistence in the environment or their potential chronic toxicity to non-target organisms, as highlighted by Ferraz and coauthors [9]. Regarding environmental considerations, results from the application of the Life Cycle Assessment revealed that oregano EO posed a great impact on human health, ecosystem integrity, and resource depletion [64,99]. The study underscores that opting for natural substances does not necessarily mean they are more environmentally friendly than synthetic products.

Although some of these products show promise, the comparison of results is quite difficult because of the variability of EOs chemical composition, the variety of concentrations used and exposure times, the heterogeneous methods employed to evaluate their antimicrobial effectiveness and the infrequent application of statistical analysis to substantiate the findings [16,87]. Future research should adhere to standardized protocols to enable more comprehensive comparisons across studies and facilitate the ranking of plant-derived extracts based on their safety profiles.

Moreover, while making a scientific comparison with conventional biocides is challenging, some EOs exhibit significant antimicrobial activity but their efficacy tends to be lower than that of traditional biocides, especially in outdoor settings where complex biofilms are present, and they often require repeated applications to be effective. Additionally, the effects of these substances on cultural heritage materials warrant more thorough investigation, as highlighted in Section 4, since certain EOs have been shown to modify the color, texture, or chemical composition of such materials.

Results described in Section 5 demonstrate that further in vitro and in vivo research is necessary to thoroughly examine the safety of EOs, especially regarding their impact on mammalian and other cell health, their interactions with particular biomolecules, and the underlying mechanisms by which they act [87]. The lack of regulation and standardization in the production and marketing of these products makes it difficult to determine the appropriate doses and concentrations for their safe use [98]. The way EOs are marketed is often unclear. They can be sold individually or as blends, and they may be categorized as medicines, food additives, dietary supplements, cosmetics, medical devices, or biocides. Their classification—and the associated safety considerations—raises important public health issues [94]. Currently, EOs are assigned a regulatory status according to their intended use, meaning their marketing category determines how they are distributed. As a result, they appear in a wide range of products, each offering different levels of safety and efficacy assurance, even though the underlying EO is identical. This situation creates a risk that EO-based products not classified as medicines may be sold without adequate guidance or warnings [82]. Evaluation of the toxicity of EOs is currently providing new knowledge of their toxic action. The whole aspect of safety is now being rigorously reviewed, and new European regulations will likely impede the sale and usage of many EOs [102].

Embedding EOs in various carrier materials represents an innovative and advanced approach to improve their stability and efficacy. Their practical use is often limited by their high volatility, hydrophobicity, and susceptibility to degradation from light, heat, and oxygen exposure [51]. These factors make EOs vulnerable to breakdown, resulting in diminished biological activity [103]. However, many authors indicate that incorporating them into different carrier materials allows even low amounts to be efficiently antimicrobial. Despite these approaches being developed, other challenges remain. Their poor water solubility and strong, persistent odor can pose additional inconveniences for restorers. The duration and costs associated with restoration treatments using EOs may also limit their widespread adoption.

In summary, the application of Eos in cultural heritage conservation is still at an early stage, facing many difficulties. More systematic long-term follow-up of treatment procedures and consideration of regrowth rates after interventions are essential. Continued research, technological development, and systematic evaluation are required to ensure that these natural products can be used safely and reliably in the protection and restoration of cultural heritage.

Finally, while treatments for biodeterioration are part of standard restoration practices, challenges remain in developing and establishing effective techniques. It may be opportune to initiate a discussion on a topic suggested by Berti and coauthors [64], namely, the consideration of a non-treatment option.

## Figures and Tables

**Table 1:** Botanical name of plant source and common names of the EOs reported in the reviewed papers. EOs extracted from plants marked with an asterisk are classified by the U.S. FDA as “Generally Recognized as Safe” (GRAS), in accordance with 21 CFR Part 182.

Scientific Name	Synonym	Common Name
*Allium sativum* L.		Garlic
*Boswellia* spp.		Frankincense
*Calamintha nepeta* (L.) Savi		Calamint
* *Cinnamomum cassia* (L.) J.Presl		Cassia
* *Cinnamomum verum* Presl	*C. zeylanicum* Blume	Cinnamon
*Citrus aurantium* L. ssp. *amara* Engl.	*Citrus aurantium* L. ssp. *aurantium* L.	Bitter orange
* *Citrus limon* (L.) Burm. F.		Lemon
*Crithmum maritimum* L.		Sea fennel
*Cyanus segetum* Hill.		Cornflower
* *Cymbopogon citratus* (DC.) Stapf		Lemongrass
*Eucalyptus globulus* Labill.		Eucalyptus
* *Foeniculum vulgare* Mill.		Fennel
*Glycyrrhiza glabra* L.		Liquorice
*Grindelia robusta* Nutt.		Gumplant
*Hamamelis virginiana* L.		Witch hazel
*Lavandula angustifolia* Mill.		English lavender
* *Lavandula latifolia* Medik.		Spike lavender
*Lavandula stoechas* L.		French lavender
*Lavandula viridis* L’Hér.		Green lavender
*Melaleuca alternifolia* Maiden & Betche		Tea tree
* *Melissa officinalis* L.		Lemon balm
* *Mentha piperita* L.		Peppermint
*Mentha pulegium* L.		Pennyroyal
*Mentha suaveolens* Ehrh.	*Mentha rotundifolia* var. *suaveolens* (Ehrh.) Briq.	Apple mint
*Monarda citriodora* Cerv. ex Lag.		Lemon bergamot
*Monarda dydima* L.		Scarlet beebalm
*Monarda fistulosa* L.		Wild bergamot
* *Nigella sativa* L.		Black cumin
* *Ocimum basilicum* L.		Basil
*Origanum vulgare* L.		Oregano
*Origanum vulgare* L. subsp. *hirtum*		Greek oregano
*Origanum vulgare* L. subsp. *viridulum* (Martrin-Donos) Nyman	*O. heracloticum* L.	Green oregano
* *Pelargonium graveolens* L’Hér.		Geranium
*Pinus cembra* L.		Pine tree
* *Rosmarinus officinalis* L.		Rosemary
* *Salvia officinalis* L.		Sage
*Satureja montana* L.		Winter savory
*Satureja thymbra* L.		Pink savory
*Syzygium aromaticum* (L.) Merr. et L.M. Perry	*Eugenia caryophyllata* Thunb.	Clove
*Thymbra capitata* (L.) Cav.	*Thymus capitatus* (L.) Hoffmanns. & Link, *Coridothymus capitatus* (L.) Cav.	Conehead thyme
*Thymus mastichina* (L.) L.		Mastic thyme
* *Thymus serpyllum* L.		Wild thyme
* *Thymus vulgaris* L.		Common thyme
* *Thymus zygis* Loefl. Ex L.		White thyme

## Data Availability

Not applicable.

## Supplementary Materials

The following supporting information can be downloaded from https://www.mdpi.com/article/10.3390/gels11120978/s1, Table S1: This table presents the essential oils, the hydrolates, their concentrations, the in vitro methods for assessing the antimicrobial activity of EOs and the microbial species they targeted in the experiments. The associated references are listed in chronological order. Further details can be found throughout the text. Acronym legend: ELAV English lavender EO, LIQ liquorice alcoholic leaf extract, HAE hydro-alcoholic extract; Table S2: Overview of biocidal products reported in the reviewed literature.
