# Supplementary material for: Essential Oils and Cultural Heritage Conservation: Are They Safe, Environmentally Friendly, Sustainable, and Negligibly Toxic?
Source: Gels. 2025 Dec 5;11(12):978. doi: 10.3390/gels11120978 (PMC12733239; doi:10.3390/gels11120978)
Supplement: Supplementary file 1 [file gels-11-00978-s001.zip › gels-3928344-supplementary.pdf]

Table S1. This table presents the essential oils, the hydrolates, their concentrations, the *in vitro* methods for assessing the antimicrobial activity of EOs and the microbial species they targeted in the experiments. The associated references are listed in chronological order. Further details can be found throughout the text. Acronym legend: ELAV English lavender EO, LIQ Liquorice alcoholic leaf extract, HAE hydro-alcoholic extract.

**Essential oils, hydrolates and extracts (plant source)**	**Concentration**	**Microorganisms**	**Methods**	**References**
EO and hydro-alcoholic extracts of basil	15 µL	*Aspergillus* sp., *Mucor* sp., *Penicillium* sp.	Disc diffusion	[34]
English lavender, oregano, rosemary	0.1-2.0 µLmL^{-1} for oregano, 10.0-100.0 µLmL^{-1} for the other two EOs	*Aspergillus niger*, *A. ochraceus*, *Bipolaris spicifera*, *Epicoccum nigrum*, *Penicillium sp.*, *Trichoderma viride*	Evaporation of EOs from filter paper discs	[11]
Tea tree EO, calamint and garlic extracts	100%, 50%, 25%, 12.5%	*Bacillus subtilis*, *Micrococcus luteus*. *Aspergillus* spp., *Penicillium chrysogenum*	Disc diffusion, agar well plate diffusion and microdilution in well plate	[43]
Hydrolates of apple mint, bitter orange, conehead thyme, cornflower, Greek oregano, green oregano, gumplant, lemon balm, lemon bergamot, rosemary, sage, scarlet beebalm, wild bergamot, winter savory, witch hazel	Diluted at a 1:2 ratio in gels	*Aspergillus sydowii*, *Cladosporium sphaerospermum*, *Penicillium chrysogenum*	Microdilution. Pieces of paper inoculated with spores, placed in contact with Gellan and hyrolates, then placed on solid agar	[53]
Cinnamon	5.625 µLmL^{-1} for fungi, and 22.5 µLmL^{-1} for bacteria	*Aspergillus niger*, *Penicillium funiculosum*, *Trichodema viride*. *Bacillus megaterium*, *Pseudomonas fluorescens*, *Streptomyces rutgersensis*	Evaporation of EOs	[12]
Common thyme, clove, geranium	0.25, 0.5, 0.75, 1 µLmL^{-1}	*Aspergillus awamori*, *A. flavus*, *A. niger*, *A.oryzae*, *A. tamari*, *A. terreus*, *A. ustus*, *A.wentii*, *Curvularia clavate*, *Fusarium oxysporum*, *Mucor fuscus*, *Penicillium citrinum*, *P. glabrum*, *P. oxalicum*, *Rhizopus oryzae*, *Stemphylum vesicarium*	Disc diffusion	[13]
Cinnamon, common thyme, wild thyme	3%, 1%, 0.7%, 0.5%	*Aspergillus japonicus*, *Chaetomium* sp., *Fusarium* sp., *Stachybotrys chartarum*	Disc diffusion (modified)	[14]
Basil, fennel, lemon, rosemary, sage	1.56-100 µLmL^{-1}	*Alternaria alternata*, *A.tenuissima*	Poisoned food	[41]
Common thyme, oregano	50%, 25%, and 12.5% (v/v)	*Aspergillus flavus*	Disc diffusion, evaporation of EOs	[66]
English lavender EO, Liquorice alcoholic leaf extract	ELAV (5% v/v), LIQ (10 and 30% v/v)	*Leptolyngbya* sp., *Scytonema julianum*, *Symphyonemopsis* sp. *Actinobacteria*, *Bacteroidetes*, *Proteobacteria*	Well plate	[39]
Lemon, common thyme	5%, 10%, and 15% in ethanol 70% (v/v)	*Aspergills niger, Fusarium* solani, *Penicillium cyclopium*	Disc diffusion	[16]
Oregano, pink savory	0.1, 0.2 and 0.5% (v/v)	*Bacillus* sp., *Paenibacillus* sp., *Stenotrophomonas* sp. *Cladosporium* sp., *Clonostachys* sp., *Fusarium* sp., *Penicillium* sp.	Well plate	[17]

Common thyme, sea fennel	50% and undiluted	*Bacillus* sp., *Georgenia* sp., *Ornithinibacillus* sp., *Streptomyces* sp.	Disc diffusion	[18]
Clove, oregano	Diluted with ethanol 70% in the ratio 2:1	*Bacillus* sp., *Penicillium* sp.	Disc diffusion and evaporation of EOs	[19]
Calamint, common thyme, oregano in hydrogels	1-2%	*Bracteacoccus minor*, *Chlorella* sp., *Stichococcus bacillaris*. *Aphanocapsa* sp., *Isocystis* sp., *Leptolyngbya cebennensis*	Stone samples inoculated with microorganisms	[37]
Calamint, common thyme, French lavender, green lavender, mastic thyme, rosemary, sage	5μL of undiluted EOs	*Aspergillus niger*, *Cladosporium* spp., *Exophiala* sp., *Fusarium oxysporum*, *Penicillium* spp. *Rhodotorula* sp. *Bacillus* sp., *Arthrobacter* sp.	Disc diffusion	[49]
Common thyme	12.5%, 25%, 50%, and 100% (v/v)	*Bacillus* sp., *Streptococcus* sp. *Aspergillus* sp., *Penicillum* sp.	Disc diffusion and agar well plate diffusion	[20]
Common thyme, oregano	5.63 and 7.5 $\mu L mL^{-1}$	*Alternaria alternata*. *Staphylococcus epidermidis*, *Rhodotorula mucilaginosa*	Well plate, tube dilution test and disc diffusion	[21]
Rosemary hydro-alcoholic extract	0.78, 1.2, 1.56 mg/mL	*Aspergillus clavatus*, *Penicillium chrysogenum*. *Arthrobacter globiformis*, *Bacillus cereus*, *B. thuringiensis*	Disc diffusion	[42]
Bitter orange hydrolate and cinnamon EO	28 $\mu L/cm^2$	*Alternaria alternata*, *Aspergillus niger*, *Aureobasidium pullulans*, *Chaetomium globosum*, *Cladosporium cladosporioides*, *Penicillium citrinum*	Disc diffusion, agar well plate diffusion and microdilution in well plates	[45]
Black cumin, clove, common thyme, geranium, lavender, lemongrass	0.1, 0.25, 0.5, 1, 2 $\mu L mL^{-1}$	*Pseudomonas protegens*, *P. putida*, *Serratia odorifera*. *Alternaria alternata*, *Aspergillus flavus*, *A. niger*, *Cladosporium halotolerans*, *Penicillium crustosum*, *Trichoderma viride*	Poisoned food	[22]
Cinnamon, scarlet beebalm, wild bergamot EOs in hydrogels. Hydrolates of bitter orange, lemon bergamot, scarlet beebalm	2-0.06 % v/v and 50-1.6 % v/v respectively for EOs and hydrolates	*Alternaria alternata*, *Aspergillus niger*, *Aureobasidium pullulans*, *Chaetomium globosum*, *Cladosporium cladosporioides*, *Penicillium citrinum*	Well plate	[23]
Common thyme and English lavender encapsulated within an alginate hydrogel	0.1% (v/v)	*Brasilonema* sp., *Leptolyngbya* sp., *Oculatella subterranea*, *Scytonema julianum*, *Symphyonemopsis* sp.	Direct application on microorganisms	[48]
Common thyme (a) or thymol (b) in hydrogels	(a) 0.25 % or 0.1 %, (b) 0.18 % or 0.07 %	*Leptolyngbya* sp., *Oculatella subterranea*, *Scytonema julianum*	Stone samples inoculated with microorganisms	[50]
Clove, oregano	7.5%(v/v) in water, with Tween 20 and Span 20 as emulsifiers	*Acremonium*-like fungus, *Cladosporium* sp., *Fusarium oxysporum*, *Mortierella* sp. *Rhodococcus* sp., *Streptomyces avidinii*	Model samples of wall paintings inoculated with microorganisms	[44]
EO and hydro-alcoholic extract (HAE) of oregano	50% EO and 100% HAE	*Bacillus* sp., *Streptomyces* sp. *Terribacillus* sp. *Alternaria* sp., *Aspergillus* sp., *Chaetomium* sp.	Disc diffusion and agar well plate diffusion	[25]
Mixture of oregano, lemongrass and peppermint in ratio 1:1:1	0.78%	*Aspergillus fumigatus*, *Cladosporium cladosporoides*, *Penicillium chrysogenum*	Disc diffusion	[35]
Eucalyptus, lemongrass, oregano, peppermint, rosemary	0.78 and 6.75%	*Aspergillus fumigatus*, *Cladosporium cladosporoides*, *Penicillium chrysogenum*	Disc diffusion	[35]

Fennel, green lavender, mastic thyme, pennyroyal	Undiluted	*Aspergillus versicolor*, *Cladosporium cladosporioides*, *Roussoella* sp., *Stagonosporopsis* sp., *Paraconiothyrium variabile*. *Cystobasidium minutum*, *Vishniacozyma globospora*. *Pseudomonas* sp., *Micobacterium* sp.	Disc diffusion	[40]
Essenzio©	10%, 20%, 50% in water, and undiluted	Cyanobacteria and algae	Model samples of wall paintings inoculated with microorganisms	[32]
Basil, cassia, clove bud, common thyme, English lavender, eucalyptus, oregano, peppermint, rosemary, tea tree	Undiluted	*Fusarium oxysporum*, *Mortierella* sp., *Bacillus cereus*, *Cupriavidus campinensis*, *Streptomyces avidinii*, *Streptomyces cirratus*	Disc diffusion	[24]
Cinnamon, eucalyptus, frankincense, geranium, lavender, lemongrass, mint, rosemary, tea tree, thyme	0.125, 0.25, 0.5, 0.75, 1 µL/mL	*Aspergillus flavus*, *A. fumigatus*, *A. niger*, *A. terreus*	Poisoned food	[27]
Fennel, green lavender, mastic thyme, pennyroyal	20% (v/v) in water	*Bacillus mobilis*, *B. wiedmannii*. *Cladosporium cladosporioides*, *Penicillium brevicompactum*	Disc diffusion and evaporation of EOs	[30]
Basil, cinnamon, common thyme, English lavender, oregano, tea tree. Essenzio©	0.5% and 5% (v/v). Essenzio© undiluted	*Acinetobacter junii*, *Aeromonas rivipollensis*, *Chryseobacterium contaminans*, *Ensifer adhaerens*, *Enterobacter quasihomaechei*, *E. sichuanensis*, *E.* sp., *Exiguobacterium mexicanum*, *Pantoea agglomerans*, *Pantoea ananatis*, *Pseudomonas alkilphenolica*, *P. chengduensis*, *P. lalkuanensis*, *P. mosselii*, *P. oryzihabitans*, *P. resinovorans*, *P. sediminis*, *P. soli*, *Serratia liquefaciens*, *S. rubidaea*, *Shigella flexneri*, *Stenotrophomonas lactitubi*. *Fusarium chlamydosporium*, *Paecilomyces lilacinus*, *Penicillium chrysogenum*, *P. citreonigrum*, *P. miczynskii*	Well plate	[29]

Table S2. Overview of biocidal products reported in the reviewed literature.

**Product Name**	**Main Components**	**Concentration / Notes**
Bioban© TM 104	Didecyldimethylammonium chloride and octyl-isothiazolinone	Antimicrobial blend
Biotin© R1+R2	R1 iodopropynyl butyl carbamate dissolved in diethylene glycol monobutyl ether; R2 n-octyl isothiazolinone and terbutryn dissolved in 2-butoxyethoxy ethanol	Antimicrobial blend
Biotin© T	Didecyldimethylammonium chloride (1), octyl-isothiazolinone (2), isopropanol (3), formic acid (4)	(1) 40-60%, (2) 7-10%, (3) 15-20%, (4) 1-2.5%
NewDes© 50	Didecyldimethylammonium chloride	50% aqueous solution
Preventol© RI 50	Alkyl benzyl dimethyl ammonium chloride (benzalkonium chloride)	~50% aqueous solution
Preventol© RI 80	Alkyl benzyl dimethyl ammonium chloride (benzalkonium chloride)	~80% aqueous solution + 2% isopropyl alcohol
